# In vivo bacteria-targeted imaging with vancomycin-based positron emission tomography and optical tracers in an orthopaedic trauma implant infection model

**DOI:** 10.1007/s00259-026-07997-x

**Published:** 2026-06-19

**Authors:** Gerbren B. Spoelstra, Lisanne M. Braams, Frank F. A. IJpma, Sjouke Piersma, Carolien S. Braams, Nicholas M. Bernthal, Kevin P. Francis, Andor W. J. M. Glaudemans, Marleen van Oosten, Ben L. Feringa, Wiktor Szymanski, Philip H. Elsinga, Jan Maarten van Dijl

**Affiliations:** 1 Department of Nuclear Medicine and Molecular Imaging, University of Groningen, University Medical Center Groningen, Hanzeplein 1, Groningen, 9713GZ The Netherlands; 2 Department of Medical Microbiology and Infection Prevention, University of Groningen, University Medical Center Groningen, Hanzeplein 1, Groningen, 9713GZ The Netherlands; 3 Department of Trauma Surgery, University of Groningen, University Medical Center Groningen, Hanzeplein 1, Groningen, 9713GZ The Netherlands; 4 Department of Orthopaedic Surgery, University of California, Los Angeles, CA 90095 USA; 5 Stratingh Institute for Chemistry, University of Groningen, Nijenborgh 4, Groningen, 9747AG The Netherlands; 6 Department of Radiology, University of Groningen, University Medical Center Groningen, Hanzeplein 1, Groningen, 9713GZ The Netherlands; 7 Department of Medicinal Chemistry, Photopharmacology and Imaging, University of Groningen, Groningen Research Institute of Pharmacy, Antonius Deusinglaan 1, Groningen, 9713AV The Netherlands; 8 Hanzeplein 1, Groningen, 9700RB The Netherlands

**Keywords:** Bacterial infection imaging, Prosthetic joint infection, Positron emission tomography, Optical imaging, ^18^F-vancomycin, Vancomycin-800CW

## Abstract

**Purpose:**

Prosthetic joint- and fracture-related infections are major postoperative complications. Early, specific, and preferably non-invasive diagnostic tools, such as bacteria-targeting imaging agents, are essential for optimal treatment, since current approaches, including white blood cell scintigraphy and [¹⁸F]FDG-PET, are accurate, but may lack specificity. Vancomycin, an antibiotic targeting Gram-positive bacteria, has been successfully applied as a molecular scaffold for bacteria-specific tracer development. This study evaluated two novel vancomycin-based PET tracers, [¹⁸F]BODIPY-FL-vancomycin and [¹⁸F]VE1-PQ-vancomycin, along with the fluorescent tracer vancomycin-IRDye800CW, for diagnosing biofilm-associated orthopaedic implant infections.

**Methods:**

We employed a preclinical in vivo model of orthopaedic implant infection, where C57BL/6 mice (*n* = 72) received stainless-steel Kirschner (K)-wire implants in the distal femur, which were inoculated with bioluminescent *Staphylococcus aureus* or *Escherichia coli* bacteria, or with PBS (sterile control). At days 13–14 post-implant infection, animals were injected with one of the vancomycin tracers or [¹⁸F]FDG (benchmark control) and underwent PET/CT or fluorescence imaging. Tracer accumulation at the infection site was quantified, followed by biodistribution analysis and bacterial load assessment.

**Results:**

Both vancomycin-based PET tracers showed significantly higher uptake in *S. aureus*-infected legs compared to sterile controls. The differences in binding between *S. aureus* or *E. coli* were less distinct. In this model, [^18^F]FDG did not distinguish K-wire infection from sterile controls. Vancomycin-IRDye800CW accumulated specifically at *S. aureus* infection sites, but not at *E. coli* infection sites or sterile controls, indicating accurate bacteria-targeted imaging.

**Conclusion:**

Vancomycin-based PET and optical tracers differentiated bacterial infection from the sterile K-wire controls in a murine model. Optical imaging distinguished Gram-positive from Gram-negative bacterial infection, whereas PET tracers did not. These preclinical findings support further investigation of vancomycin-based tracers for non-invasive imaging of prosthetic joint and fracture-related infections.

**Supplementary Information:**

The online version contains supplementary material available at 10.1007/s00259-026-07997-x.

## Introduction

Biomaterial-associated infections (BAIs) are challenging complications following musculoskeletal surgery, and they pose a significant burden both for patients and the healthcare system [1–4]. In orthopaedic surgery the incidence of prosthetic joint infections (PJIs) is 1–2%, and in trauma surgery the incidence of (open) fracture-related infections (FRIs) amounts up to 30% [2, 5, 6]. Implanted biomaterials inherently facilitate bacterial colonisation. This may lead to chronic, treatment-resistant infections due to biofilm formation on the implants [7]. Such biofilms are composed of sessile bacterial communities embedded in a self-produced extracellular polymeric substance (EPS) that shelters the bacteria from the host’s immune defences and antibiotic treatments [7, 8].

The clinical presentation of FRIs and PJIs varies from acute purulent drainage to subtle signs. The latter complicates the differentiation between bacterial infection and sterile inflammation [9, 10]. Sterile inflammation may be caused by trauma, including bone fracture and soft tissue damage, or by an implanted foreign body [7]. However, inflammation is also part of the healing process that supports fracture repair and tissue regeneration. The current diagnosis of bacterial infections relies on invasive microbiological sampling and culturing, which is not always feasible and prevents a definitive diagnosis of infection before or during surgery. This uncertainty complicates decision-making regarding the extent of debridement, implant retention or removal, and the initiation and selection of appropriate antibiotic therapy. Moreover, it delays targeted treatment [11]. Pending a microbiological diagnosis, broad-spectrum antibiotic therapy is often started empirically, thereby increasing the risk of antibiotic resistance development [2]. If this results in treatment-resistant infection and non-union of bones, amputation may be the only remaining treatment option [2, 6].

Diagnostic information concerning infection can be acquired through anatomical and functional imaging modalities, such as radiography, computed tomography (CT), positron emission tomography (PET) and single-photon emission computed tomography (SPECT) [2, 10, 12]. White blood cell (WBC) scintigraphy and PET imaging with [^18^F]fluorodeoxyglucose ([^18^F]FDG) are most commonly used in clinical practice, but they primarily detect indirect signs of infection, such as leukocyte infiltration, increased glucose metabolism, implant loosening, or impaired bone healing [2, 10]. Nonetheless, these techniques can achieve high diagnostic accuracy, particularly WBC scintigraphy for PJI and [^18^F]FDG-PET for FRI [10, 13–15]. However, each modality has inherent limitations, including reduced specificity of [^18^F]FDG-PET in low-grade infections and challenges in the early post-operative setting when distinguishing infection from normal wound healing. In addition, WBC scintigraphy is labor-intensive and requires time-consuming procedures. Consequently, differentiating bacterial infection from sterile foreign body responses or post-operative changes remains challenging in certain clinical contexts [2, 10, 15, 16].

In response to these challenges, bacteria-targeted imaging has been explored as a promising approach to diagnose infection [16]. Radioisotopes or fluorophores can be conjugated to bacteria-targeting molecules, such as antibiotics, antibodies or metabolites, to produce nuclear or optical imaging tracers [11]. Optical tracers are ideal for image-guided surgery due to real-time visualisation of infection foci [17, 18]. However, limited tissue penetration of the emitted signal (~ 1 cm), even in the near-infrared (NIR) spectrum, restricts the use of optical tracers for the detection of infections, unless paired with endoscopic imaging [19]. For the detection of deeper-seated infections, which may require whole-body imaging, bacteria-targeted PET imaging is an attractive alternative [11]. 

FRIs and PJIs are caused by Gram-positive bacteria in approximately 55–75% of cases, among which *Staphylococcus aureus* (*S. aureus*) is the most common causative pathogen [5, 6]. This makes vancomycin, which binds to the d-Alanyl-d-Alanyl (d-Ala-d-Ala) motif in the Gram-positive bacterial cell wall, a candidate targeting molecule for the development of specific tracers [20]. Previously, the NIR optical imaging tracer vancomycin-IRDye800CW (vancomycin-800CW; Figure S1) has proven effective in differentiating Gram-positive from Gram-negative bacterial infections or inflammation in murine myositis and spinal implant infection models [21, 22]. Vancomycin-800CW has also proven effective in a human *post-mortem* PJI model, and the detection of biofilms on extracted osteosynthesis devices from patients with FRI [19, 23]. Furthermore, we have recently developed two vancomycin-based PET tracers: [^18^F]BODIPY-FL-vancomycin and [^18^F]VE1-PQ-vancomycin (Figure S1) [24–26]. Their in vivo evaluation in a murine *S. aureus* myositis model showed that both PET tracers accumulated at sites of infection, with minimal uptake in healthy animals and at sites of sterile inflammation. This suggests clinical specificity, though direct evidence for specific binding to Gram-positive bacteria in vivo remains to be established [25]. Despite these preclinical advances, in vivo detection of FRI and PJI with vancomycin-800CW or vancomycin-based PET tracers was not yet evaluated. 

The Kirschner (K)-wire implant infection model is a well-established murine model for the assessment of PJI [27–29], and for evaluation of the efficacy of antimicrobial agents [28, 30–34]. In this model, a K-wire is inserted into the femoral intramedullary canal and inoculated with either bioluminescent *S. aureus*, *Escherichia coli* (*E. coli*), or sterile phosphate-buffered saline (PBS). The course of infection can then be monitored through bioluminescence imaging (BLI) over a period of up to 14 days [28, 30–34]. In the present study, we implemented the K-wire infection model to assess the in vivo specificity of [^18^F]BODIPY-FL-vancomycin and [^18^F]VE1-PQ-vancomycin for the detection of implant-associated infection caused by *S. aureus*. The performance of these PET tracers was compared with that of vancomycin-800CW and the metabolic tracer [^18^F]FDG.

## Materials and methods

### Bacteria

The *S. aureus* strain Xen36 and the *E. coli* strain Xen14 were used for the present studies. *S. aureus* Xen36 is based on the American Type Culture Collection (ATCC) strain 49525 [35], and *E. coli* Xen14 is based on the Weihenstephan Microbial Strain Collection (WS) strain 2572 [36]. Both strains have been genetically modified to express the *lux* operon from *Photorhabdus luminescens* for blue-green bioluminescence (490 nm) by metabolically active bacteria, and both carry a kanamycin resistance marker [27, 35, 37]. *S. aureus* was cultured in tryptic soy broth (TSB, Oxoid, Hampton, USA), and *E. coli* Xen14 in lysogeny broth (LB, BD Difco, Franklin Lakes, USA). Both media were supplemented with 250 µg/mL kanamycin (Sigma Aldrich, Saint Louis, USA) unless noted otherwise.

### Preparation of bacterial cultures

-80 °C glycerol stocks of *S. aureus* Xen36 and *E. coli* Xen14 were plated on LB agar (LBA, BD Difco) or tryptic soy agar (TSA, Oxoid) and incubated at 37 °C for 24 h. A single colony was used to prepare an overnight culture, which was incubated at 37 °C and 250 revolutions per minute (rpm). Freshly inoculated cultures had a starting optical density at 600 nm (OD_600_) of 0.05 and were grown in about 3 h to the mid-logarithmic growth phase (OD_600_ ~ 0.5–1). Bacteria were collected by centrifugation (10 min, 3000 relative centrifugal force (rcf)), washed twice in 1 mL PBS, centrifuged between washes (1 min, 5000 rpm), and resuspended in 2.5 µL PBS at ~ 1.25 · 10^4^ (*S. aureus*) or 1.25 · 10^5^ (*E. coli*) colony-forming units (CFUs). CFU counts of the bacterial inoculums were approximated by OD_600_ and confirmed by plating on Mueller Hinton agar (MHA, Oxoid) directly upon preparation.

### Mice

Eight-week-old, 25 g C57BL/6 wild-type male mice (Charles River, Wilmington, USA) were housed with 3 mice per cage under a 12 h light and dark cycle with food and water provided *ad libitum*. Animal condition was checked daily. Animal welfare was monitored weekly in the period leading up to the K-wire surgery, daily during the first 4 days after the surgical K-wire placement, and every third day thereafter. Particular attention was paid to animal behaviour, locomotion, and animal weight. All animal procedures were performed in adherence with national and local regulations and were approved by the University Medical Center of Groningen (UMCG) animal ethics board (permit number 2114768-01-004).

### Kirschner wire infection study design

Animals were randomly divided into equally sized groups to receive either the vancomycin-based PET tracers [^18^F]BODIPY-FL-vancomycin or [^18^F]VE1-PQ-vancomycin, or the fluorescent tracer vancomycin-800CW, or [^18^F]FDG. Per tracer (*n =* 4) three groups of 6 animals were used: in one group the inserted K-wire was infected with *S. aureus* Xen36 (*n* = 6), in one group the K-wire was infected with *E. coli* Xen14 (*n* = 6), and in one group the inserted K-wire was treated with sterile PBS to obtain sterile controls (‘sterile K-wire’; *n* = 6); (*n =* 18 per tracer, *n* = 72 in total). The untreated contralateral hind leg of each mouse (i.e. without an inserted K-wire) served as healthy control (‘no K-wire’). After injection with one of the four tracers, animals were imaged with a microPET/CT imaging system or a fluorescence imaging device as described below. Due to the nature of the study, the investigators could not be blinded to the (infection) status of the animals.

### Kirschner wire surgical procedures

K-wire implant surgery was performed as previously described [27, 29]. Animals were anaesthetised using isoflurane (induction 5%, maitenance 1.5–2.5%), an buprenorphine (0.1 mg/kg) was administered subcutaneously 15 min prior to incision, which was once repeated 8 h post-surgery if deemed necessary based on animal behaviour. A midline skin incision was made over the left knee, the fascia was incised and the parapatellar tendons were sharply dissected to displace the quadriceps-patellar complex to gain access to the distal femur. The femoral intermedullary canal was prepared by manual reaming with, subsequently, 25 gauge (G) and 23 G needles. An initial inoculum of 1.5 µL bacterial suspension, or 1.5 µL sterile PBS, was pipetted into the canal using an ultrafine 10 µL micropipette tip. Next, a surgical stainless-steel K-wire (⌀ = 0.8 mm, l = 6 mm, Stryker European Operations Ltd, Cork, Ireland) was placed in a retrograde fashion, protruding slightly into the knee-joint space. A second inoculum (1 µL) was then pipetted into the joint space, after which the quadriceps-patellar complex was reduced to midline and the surgical site was closed with vicryl 5 − 0 sutures (Ethilon, Raritan, USA).

### Confirmation of infection using bioluminescence imaging

On postoperative days 4, 10 and 13/14, bioluminescence imaging was performed on anaesthetised animals to quantify the bacterial burden using a LAGO imaging system (Spectral Instruments Imaging, Bruker, USA). Images were acquired at 150 s and 300 s exposure time. Regions of interest (ROIs; 1.8 cm × 1.3 cm, ellipse) were drawn, and measurements were quantified using Spectral Instruments Aura software (version 4.5.0). Data are expressed as mean radiance.

### Vancomycin-based imaging agents

The optical tracer vancomycin-IRDye800CW was prepared as previously described [38], and the PET tracers [^18^F]BODIPY-FL-vancomycin and [^18^F]VE1-PQ-vancomycin (Figure S1) were also synthesised as previously described [24, 39]. [^18^F]FDG was produced in-house under GMP conditions for clinical application and used without further processing from stocks of 200 MBq in 5 mL 0.9% saline. Both vancomycin-based PET tracers were formulated using an Oasis HLB cartridge in absolute EtOH. The concentration of EtOH was brought below 10% using a 0.9% saline solution. Net injected doses (IDs) were 1.73 MBq ± 0.34, 0.94 ± 0.25, and 2.08 ± 0.32 for [^18^F]BODIPY-FL-vancomycin, [^18^F]VE1-PQ-vancomycin, and [^18^F]FDG, respectively. The molar activity of [^18^F]BODIPY-FL-vancomycin and [^18^F]VE1-PQ-vancomyin was previously determined at 5.35 ± 3.91 GBq/µmol and 415 ± 210 GBq/µmol, respectively [24].

### Fluorescence imaging

A 200 µM stock solution of vancomycin-800CW was prepared from a lyophilised sample. On postoperative day 13, mice were anaesthetised and injected retro-orbitally with 50 µg (~ 1 mg/kg) of the tracer. At 32 h post-injection, animals were re-anaesthetised, and hind leg fur was removed for fluorescence imaging (FLI) using a LAGO imaging system. After scanning, mice were euthanised by cervical dislocation, and hind legs and relevant organs were collected for ex vivo analysis. To mimic intraoperative imaging, the hind leg skin was removed, followed by repeat FLI. Data were analysed using Aura software with elliptical ROIs (1.8 × 1.3 cm, ellipse) drawn around hind legs and organs. Biodistribution of the tracer was assessed using an Amersham Typhoon Biomolecular Imaging System (Cytiva, USA), with freehand ROIs quantified via ImageJ (v1.54d).

### Positron emission tomography and computed tomography

On post-operative days 13 or 14, the tracer was administered retro-orbitally with tracer uptake times of 60 min for [^18^F]BODIPY-FL-vancomycin and [^18^F]FDG, and 120 min for [^18^F]VE1-PQ-vancomycin. Animals were not fasted prior imaging to maintain consistent experimental conditions across tracer groups. This was necessary, because tracer usage was determined on the day of imaging based on tracer availability. PET/CT imaging was performed using a Siemens Inveon microPET/CT (Siemens, Erlangen, Germany), with up to three animals scanned simultaneously. Before PET acquisition, bladders were emptied via 25 G needle puncture. PET data was acquired over 20 min and reconstructed in a single frame using OSEM2D-Z1-SC-256 reconstruction parameters. Two CT scans were performed; one for attenuation correction and another for anatomical co-registration. Images were analysed with Amide (v 1.0.6). Cylindrical ROIs (⌀ = 3 mm, l = 10 mm) were drawn on the PET scans, referencing CT images to delineate the anatomical location of the K-wire. For each animal, two ROIs were drawn: one covering the K-wire-implanted femur, and one covering the contralateral femur without K-wire as an internal control (Figure S2). Standardised uptake values (SUVs) were calculated, corrected for ID, decay and body weight. Three-dimensional (3D) visualisation of PET/CT scans was conducted using the Python-based tool PyVIVIPET 1.0 developed for this purpose (https://github.com/PyVIVIPET/PyVIVIPET). Following imaging, mice were euthanised by cervical dislocation and hind legs and relevant organs were collected for ex vivo analysis using a gamma counter (Wizard2, PerkinElmer, USA). Radioactivity was corrected for decay, weight, animal weight and ID, and expressed as either absolute counts per second (cps), or %ID/g.

### Quantification of bacteria in collected murine tissues

Bacterial presence at termination was assessed by dilution plating of femur, tibia, and K-wire samples. After radioactivity quantification via gamma counting, tissue samples were homogenised (Bertin, Montigny-le-Bretonneux, France) for 3 × 30 s at 5000 rpm. Before homogenisation, the K-wire was removed, placed in 0.5 mL Mueller Hinton broth (MHB, Oxoid) with 0.3% Tween, and vortexed for 5 min to dislodge adherent bacteria. Homogenised bone samples were briefly centrifuged (20 s, 5000 rpm), and 20 µL of supernatant was serially diluted and plated on Mueller Hinton agar (MHA) in 5 µL spots. After drying, plates were incubated at 37 °C overnight. If no growth appeared after 24 h, plates were incubated for an additional 24 h before reassessment. Bacterial burden was ultimately expressed as CFUs/mL.

### Scanning electron microscopy

Bacterial biofilm formation on K-wire implants was confirmed by scanning electron microscopy (SEM). K-wires from *S. aureus-* (*n =* 2) or *E. coli-* (*n =* 2) infected animals were collected post-mortem from crushed bone, preserving the intra-articular biofilm. Samples were fixed in 4% paraformaldehyde (PFA) for 24 h at room temperature (RT), washed twice with sterile PBS, and stored at 4 °C. Secondary fixation was done with 2% glutaraldehyde (30 min, RT), followed by incubation in 1% osmium tetroxide (OsO₄) in 0.1 M cacodylate buffer (1 h, RT), and rinsing with water. Samples were dehydrated through graded ethanol desiccation (30%–50%–70%–100%, 10 min each) followed by two incubation steps in 100% ethanol (20 min, 30 min). Subsequently, they were treated with 1:1 ethanol: tetramethyl silane (TMS) (10 min), and then with 100% TMS (15 min). Lastly, the K-wires were mounted on aluminium stubs using conductive carbon stickers as an adhesive, sputter-coated with gold-palladium, and imaged using a Zeiss Supra55 ATLAS/EDX SEM (Carl Zeiss Microscopy GmbH, Oberkochen, Germany).

**Fig. 1:**
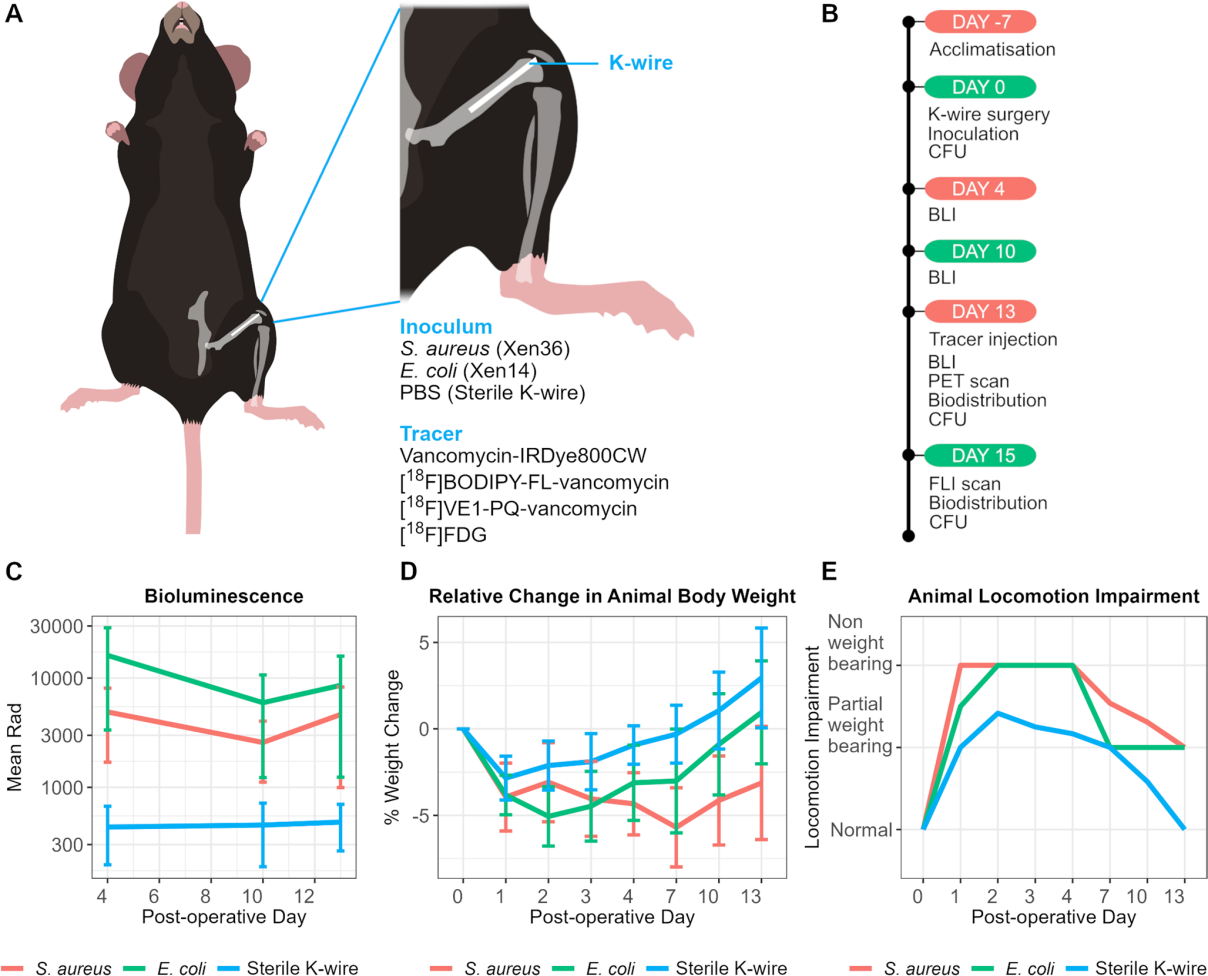
K-wire infection model set-up, infection course and animal welfare. **A** A K-wire was implanted into the femoral intramedullary canal and inoculated with bioluminescent *S. aureus*, *E. coli*, or PBS as ‘sterile K-wire’ control (*n* = 6/group). Mice received vancomycin-800CW, [¹⁸F]BODIPY-FL-vancomycin, [¹⁸F]VE1-PQ-vancomycin, or control tracer [¹⁸F]FDG (*n* = 18/tracer). **B** Experimental timeline: mice acclimated for 7 days; infection was induced on day 0; BLI was performed on days 4, 10, and 13; tracers were administered on days 13 or 14; imaging and tissue collection followed on the same day (PET tracers) or on day 15 (vancomycin-800CW). **C** Bioluminescence over time, shown as mean radiance ± SEM. **D** Relative change in animal body weight over time, shown as mean ± SD. **E** Animal locomotion impairment over time. Locomotion was visually scored as normal (preoperative mobility); partial weight-bearing (limp with some use of the leg); non-weight-bearing (nearly no weight was placed on the affected leg)

### Statistical analyses

All data are presented as mean ± standard deviation (SD), unless otherwise stated. Data analysis was performed with R v. 4.3.1 (R core team 2022, Vienna, Austria) using R studio v. 2023.06.0. Normality was assessed using Shapiro–Wilk tests and homogeneity of variances using Levene’s test. Given the small sample sizes and potential deviations from assumptions, group differences were evaluated using Welch’s ANOVA. Pre-specified pairwise comparisons versus *S. aureus* were conducted using Welch’s t-tests with Holm correction for multiple testing. A *p*-value < 0.05 was considered statistically significant.

### Clinical trial number

Not applicable.

## Results

### Murine K-wire biofilm infection model; chronic orthopaedic trauma implant infection and recovery of animal welfare

To evaluate vancomycin-based tracers for detecting orthopaedic implant infections, a K-wire infection model was used (Fig. 1). A K-wire was surgically implanted into the femoral intramedullary canal and inoculated with bioluminescent *S. aureus* (Xen36), *E. coli* (Xen14), or PBS as a sterile K-wire control. The contralateral leg without an implanted K-wire remained untreated and served as a healthy control (Fig. 1A). Inocula of 1.25 × 10⁴ CFUs (*S. aureus*) or 1.25 × 10⁵ CFUs (*E. coli*) established persistent localised infections, as confirmed by culture of the femur, tibia and K-wire (Figure S3). Notably, the tibia was typically culture-negative, indicating that the bacteria did not spread beyond the knee joint and femur. Bioluminescence imaging (BLI) on days 4, 10, and 13 showed peak signal on day 4 for both pathogens, indicating acute infection (Fig. 1C). The subsequent dip and later increase, possibly marked a shift towards persistent chronic infection, which served as the intended infection stage for tracer evaluation. Of note, as BLI reflects metabolic activity, the signal drop may also reflect a reduced bacterial metabolism, such as occurs in biofilms, rather than bacterial death [21, 40]. To further explore this finding, bacterial biofilm formation by *S. aureus* or *E. coli* on K-wires collected on day 13 was verified using SEM in a separate experiment (Figure S4).

**Fig. 2:**
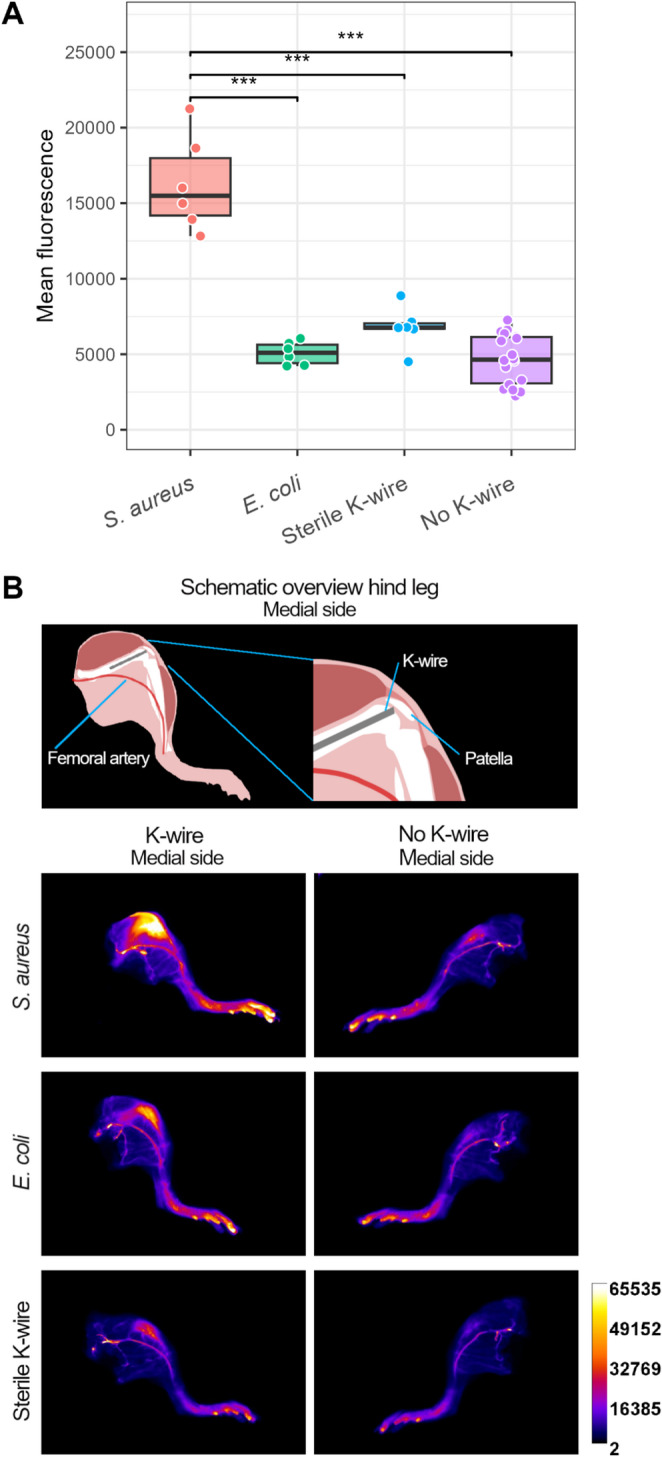
*S. aureus*-specific optical imaging of murine K-wire implant infection with vancomycin-800CW. **A** Tracer uptake in murine hind legs (*n* = 6 mice per condition). Healthy tissue from the contralateral legs was used as control for autofluorescence. Data is represented as the median with the interquartile range (IQR) with individual datapoints. Welch’s ANOVA (*p* < 0.001) followed by planned Welch’s t-tests comparing each condition to *S. aureus* with Holm correction: ***, *p* < 0.001. **B** Schematic overview of the murine hind leg with an inserted K-wire and representative FLI images (medial side) of the post-termination legs of one animal per condition. Tracer is shown to accumulate in the knee joint area of legs with an implanted K-wire, most prominently at the S. aureus infection site. The contralateral leg without a K-wire (‘no K-wire’) reveals autofluorescence of healthy tissue and the femoral artery. The images were recorded with a Typhoon Biomolecular imaging system

During the course of the experiment, animal body weight and welfare were closely monitored (Fig. 1D, E). PBS-treated animals lost 2% of their pre-surgery weight within the first 24 h post-surgery, but regained weight steadily throughout the following days. In contrast, infected animals lost ~ 5% of body weight, with the most pronounced and lasting loss in *S. aureus*-infected mice (Fig. 1D). Similarly, locomotion was impaired in all groups early on, particularly in infected mice, which did not use the operated leg for the first 4 days. Recovery was faster in the *E. coli* group, with minor residual limping, while PBS-treated mice regained full mobility (Fig. 1E).

On days 13 or 14, mice received either the optical tracer vancomycin-800CW or one of three PET tracers: [¹⁸F]BODIPY-FL-vancomycin, [¹⁸F]VE1-PQ-vancomycin, or the [¹⁸F]FDG control (Fig. 1B). Tracer uptake times were optimised based on prior investigations [21, 24, 25], and pilot data (Figure S5). Vancomycin-800CW (1 mg/kg) was imaged after 32 h, while for PET tracers, uptake times of 60 min were used for [¹⁸F]BODIPY-FL-vancomycin and [¹⁸F]FDG, and 120 min for [¹⁸F]VE1-PQ-vancomycin. The shorter uptake time of 60 min for [¹⁸F]BODIPY-FL-vancomycin was chosen because of this tracer’s faster renal clearance. Of note, hydrophilicity was previously observed to be the primary tracer characteristic affecting signal clearance from non-targeted muscle tissue and organs, while maintaining signal in the target leg [24].

**Fig. 3:**
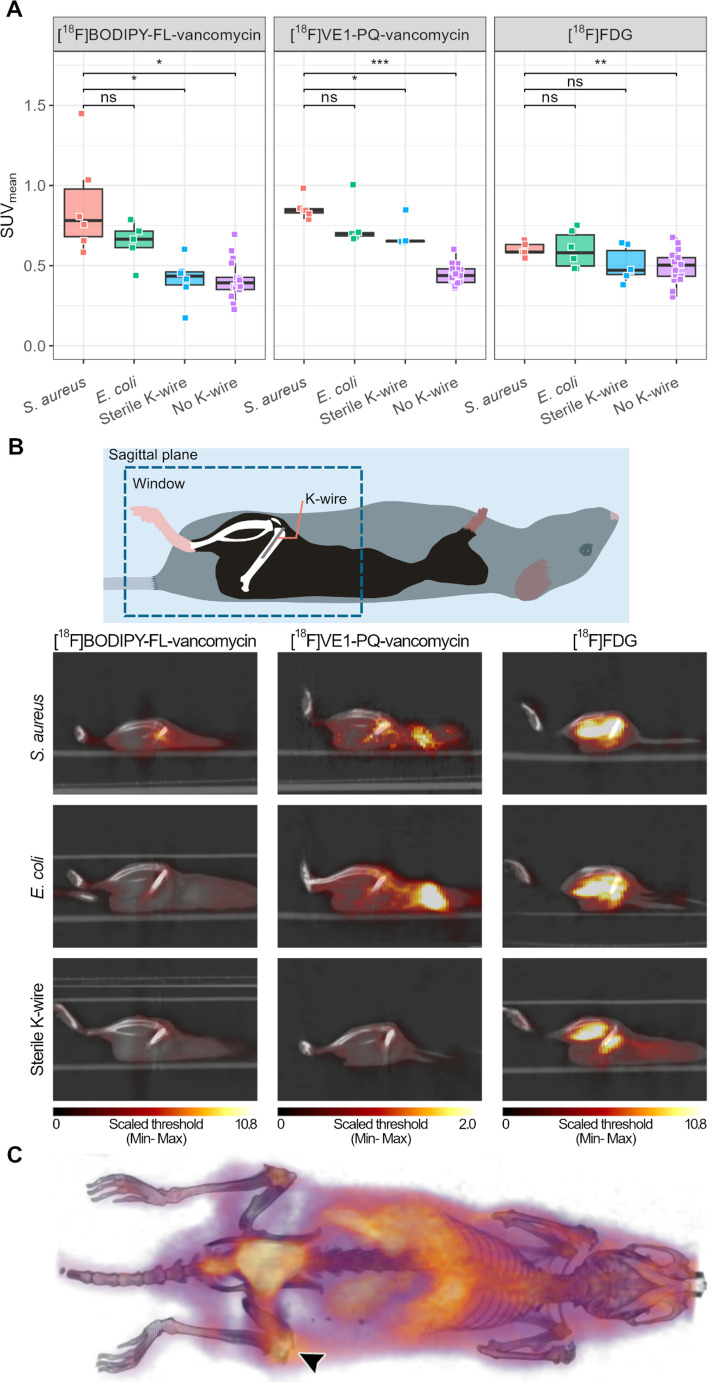
PET/CT imaging of murine K-wire implant infection with [^18^F]BODIPY-FL-vancomycin, [^18^F]VE1-PQ-vancomycin or [^18^F]FDG. **A** Tracer uptake in hind legs. The Y-axis represents the SUV_mean_, and the X-axis represents the infection status (*n* = 6 mice per condition). Healthy tissue from the contralateral legs was used as background. Data is represented as the median with the interquartile range (IQR) and individual datapoints. Statistical significance was tested by Welch’s ANOVA (*p* = 0.007) followed by planned Welch’s t-tests comparing each condition to *S. aureus* with a Holm correction: *, *p* < 0.05; **, *p* < 0.01; ***, *p* < 0.001. **B** Representative sagittal PET/CT images from one animal per condition, showing tracer uptake in the legs with an implanted K-wire. Animals are depicted in the supine position, lateral view. The blue rectangle in the schematic overview indicates the sagittal imaging plane, corresponding to the window visualized in the PET/CT images shown below. The K-wire appears as a bright white pin on the CT scan, see also Figure S2. The threshold for PET datasets of the depicted animals was set at 10.8% for [^18^F]BODIPY-FL-vancomycin and [^18^F]FDG, or 2.0% for [^18^F]VE1-PQ-vancomycin. **C** Total body visualisation of the [^18^F]BODIPY-FL-vancomycin-based PET/CT imaging of a K-wire infection by *S. aureus* (black arrow head, left knee-joint space). The animal is in supine position, anterior view. Displayed signal intensities are normalized. For a 3D visualisation, see Movie S1

### Vancomycin-800CW optical imaging allows detection of *S. aureus* orthopaedic trauma implant infection

To benchmark the recently developed vancomycin-based PET tracers, the more extensively characterised optical tracer vancomycin-800CW was included in the K-wire infection model (Fig. 2). A strong NIR signal was measured in the *S. aureus-*infected legs, which was significantly higher than the NIR signals detected in the *E. coli-*infected legs, legs with a sterile K-wire, or healthy non-infected contralateral legs without a K-wire (Fig. 2A). Furthermore, this high contrast in signal intensity was also apparent in the FLI images (Fig. 2B). To exclude a potential influence of ROI size on these measurements, ROI areas were compared across groups, showing no significant differences (Figure S6C). The tracer uptake was highest in the distal femur and knee joint of *S. aureus-*infected animals (Fig. 2). Areas surrounding the sterile K-wire in PBS-inoculated animals were visually indistinguishable from healthy control legs without a K-wire. Some autofluorescence was detectable in the femoral artery, patella and paw extremities. Furthermore, vancomycin-800CW signal was detected in the lungs, kidneys and urine (Figure S6AB), as was previously documented [21].

### Vancomycin-based PET differentiates infection from sterile conditions more effectively than [^18^F]FDG

The performance of the vancomycin-PET tracers for biofilm infection imaging was compared to both vancomycin-800CW and [^18^F]FDG. For both vancomycin-PET tracers, the highest average signal was observed in *S. aureus-*infected animals (Fig. 3A). Importantly, this signal was significantly higher than in animals with a non-infected PBS-treated (sterile) K-wire and healthy control legs (no K-wire). Nonetheless, the differences were not as pronounced as observed upon optical imaging with vancomycin-800CW, and the vancomycin-PET tracers were also found to accumulate in *E. coli*-infected legs. On the other hand, [^18^F]FDG showed comparable accumulation in the infection and sterile K-wire conditions, albeit that significantly more [^18^F]FDG signal accumulated in *S. aureus*-infected legs than in the healthy control without K-wire (Fig. 3A). Analysis of the PET/CT images revealed that both vancomycin-PET tracers were prominently localised to the knee joints and distal femur of *S. aureus*-infected animals (Fig. 3B, C, Figure S7, Movie S1). As expected for a glucose-based tracer, [^18^F]FDG was mainly localised in the leg musculature, practically overlapping with the knee joint. Post-termination biodistribution analysis of the legs and relevant organs confirmed the PET/CT findings, showing similar tracer accumulation patterns in the legs (Figure S8). The target-to-non-target (T/NT) ratios for both [¹⁸F]BODIPY-FL-vancomycin (2.54 ± 0.47) and [¹⁸F]VE1-PQ-vancomycin (1.85 ± 0.25) in *S. aureus* infection were significantly higher than in the sterile K-wire conditions (Table 1). In addition, relatively high signals were observed in the kidneys and lungs with both vancomycin-based PET tracers (Figure S8A, Table S1). Notably, [^18^F]VE1-PQ-vancomycin showed a more pronounced spleen, liver and intestinal uptake, alluding to hepatobiliary clearance alongside renal clearance. These results align with earlier findings from biodistribution studies with our vancomycin-PET tracers [25]. To approximate tracer binding at the bacterial level, we calculated the number of tracer molecules per CFU based on injected activity, molar activity, and bacterial counts (Table S2). For [¹⁸F]BODIPY-FL-vancomycin approximately 5- to 50-fold higher molecules/CFU were observed compared to [¹⁸F]VE1-PQ-vancomycin. These differences are consistent with the lower molar activity of [¹⁸F]BODIPY-FL-vancomycin and should be interpreted as an approximate measure of tracer-bacteria association.

**Table 1:** Target to non-target ratios of [^18^F]BODIPY-FL-vancomycin, [^18^F]VE1-PQ-vancomycin and [^18^F]FDG

Tracer	Condition	T/NT Mean ± SD	95% CI	*p* (Welch t-test)	*p* (Welch ANOVA)
[^18^F]BODIPY-FL-vancomycin	*S. aureus*	2.54 ± 0.47	2.05–3.03	–	**0.022**
	*E. coli*	1.91 ± 0.29	1.61–2.22	**0.022**	
	Sterile K-wire	1.79 ± 0.23	1.55–2.03	**0.018**	
[^18^F]VE1-PQ-vancomycin	*S. aureus*	1.85 ± 0.24	1.59–2.11	–	**0.016**
	*E. coli*	1.65 ± 0.18	1.46–1.84	0.143	
	Sterile K-wire	1.40 ± 0.18	1.21–1.59	**0.011**	
[^18^F]FDG	*S. aureus*	1.12 ± 0.21	0.86–1.38	–	0.490
	*E. coli*	1.15 ± 0.21	0.89–1.41	0.858	
	Sterile K-wire	1.28 ± 0.21	1.05–1.50	0.529	

Welch’s one-way ANOVA with planned Welch’s t-tests versus *S. aureus* (Holm-adjusted). Significant results (*p* < 0.05) are shown in bold. *SD*, standard deviation; *CI,* confidence interval

## Discussion

In this study, vancomycin-based bacteria-targeted imaging was evaluated for its potential to diagnose orthopaedic trauma infections using a murine K-wire implant model. Fluorescence and PET imaging in the K-wire infection model were differently implemented to explore their applicability for real-time image-guided surgery and the detection of deep-seated infections, respectively. In particular, fluorescence imaging was performed ex vivo at 32 h post-injection to simulate intraoperative conditions, while PET imaging was conducted in vivo at earlier timepoints (1–2 h) post-injection. The results show that fluorescence imaging using vancomycin-800CW effectively differentiated *S. aureus* infection from both *E. coli* infection and sterile conditions. Likewise, PET imaging with [¹⁸F]BODIPY-FL-vancomycin or [¹⁸F]VE1-PQ-vancomycin showed significantly higher tracer uptake in *S. aureus*-infected legs compared to legs with a sterile K-wire and contralateral control legs without an inserted K-wire. In contrast, we show that [¹⁸F]FDG accumulated in infected and sterile tissues alike, underscoring its limited specificity. Here it should be noted that the absence of fasting may have reduced the diagnostic performance of [¹⁸F]FDG, but this was necessary for maintaining consistent experimental conditions across tracer groups. 

The results obtained with the two vancomycin-based PET tracers, together with the biodistribution data gathered in earlier studies [24, 25], suggest that [^18^F]BODIPY-FL-vancomycin is cleared more rapidly than [^18^F]VE1-PQ-vancomycin. This may relate to a higher hydrophilicity of the [^18^F]BODIPY-FL-vancomycin tracer [24, 25]. Furthermore, tracer clearance around the K-wire was slower than in the contralateral uninfected control leg. This suggests that T/NT ratios around the implant could still improve over time. Thus, it may be worth exploring in future studies whether longer tracer incubation times will result in higher T/NT ratios at sites of infection, although this may require injection of higher tracer doses to compensate for the rapid ^18^F decay. An alternative would be to develop vancomycin conjugates with other radioisotopes, such as ^89^Zr, which has a half-life of 3.27 days. This would allow imaging over several days after tracer administration. To label vancomycin with ^89^Zr, the BODIPY or VE1-PQ groups would need to be replaced with appropriate chelators for radiometal coordination. Notably, at the time of imaging, the bacterial burden in the murine K-wire infection model was generally below 10⁶ CFUs, which may approach the detection limit for vancomycin-based (PET) tracers [21, 22]. It will thus be important to determine in future studies how the detection of bacterial biofilms on infected implants with the aid of such tracers is affected by the interplay between blood flow, tissue perfusion, tracer binding by the bacteria and tracer clearance.

Vancomycin-800CW demonstrated clear differentiation between *S. aureus*, *E. coli* and sterile K-wire conditions. On the other hand, [¹⁸F]BODIPY-FL-vancomycin and [^18^F]VE1-PQ-vancomycin only distinguished infection from sterile conditions upon PET imaging. For yet unknown reasons, these tracers did not differentiate between infections caused by the Gram-positive bacterium *S. aureus* and the Gram-negative bacterium *E. coli*. The observed differences in tracer performance, as well as the limited ability to discriminate between bacterial species, may be related to a combination of relatively low bacterial loads and slow tracer clearance from the tissues. In addition, non-specific tracer accumulation driven by inflammation cannot be excluded as a contributing factor, since capillary leakage and vasodilation associated with the inflammatory response may result in enhanced tracer extravasation and prolonged retention at infection sites, irrespective of bacterial species. These findings can be placed in the context of our previously reported findings in a murine myositis model [25]. In the myositis model, an acute infection of the hind leg thigh muscle was induced by intramuscular injection of either *S. aureus* or *E. coli* with a high bacterial burden, whereas sterile inflammation was induced by injection of Cytodex beads. Subsequently, the animals were injected with tracer 48 h post-infection. In this model, we showed that [^18^F]BODIPY-FL-vancomycin and [^18^F]VE1-PQ-vancomycin were able to clearly differentiate between infection and inflammation, and [^18^F]VE1-PQ-vancomycin additionally differentiated *S. aureus* from *E. coli* [25].

Previous studies have shown that vancomycin-based tracers are highly promising for the diagnosis of soft-tissue infections in vivo, and implant-related infections ex vivo [19, 21, 23, 25], thereby underlining the great potential of bacteria-targeted infection imaging. Particularly, the application of vancomycin-based tracers in fluorescence imaging allows for real-time intraoperative detection of infection foci, potentially providing essential information to guide surgeons in the debridement of infected implants and tissues [24–26]. On the other hand, bacteria-targeted PET imaging can potentially be utilised to diagnose infections and, if so, assess their extent. Importantly, this would reduce unnecessary surgeries or suboptimal treatment. It should be noted that vancomycin-based tracers target the bacterial cell wall and therefore also bind to non-viable Gram-positive bacteria, as demonstrated in our previous in vitro studies [25]. While this may limit their use for monitoring a therapeutic response, it could also contribute to sustained signal at infection sites. In addition, the structural modifications of vancomycin that we have implemented in our studies reduce its antibacterial activity. As previously shown, [^18^F]BODIPY-FL-vancomycin exhibited an increased minimal inhibitory concentration compared to vancomycin, whereas [^18^F]VE1-PQ-vancomycin did not show any measurable antibacterial activity, indicating that these tracers are unlikely to exert meaningful antibiotic pressure in vivo [24]. The same was found to be true for vancomycin-800CW [38]. Another important consideration is whether vancomycin-based tracers are applicable across different bacterial strains, including those with varying antibiotic resistance profiles. In vitro experiments with clinically relevant species demonstrated selective binding of both [^18^F]BODIPY-FL-vancomycin and [^18^F]VE1-PQ-vancomycin to various different Gram-positive bacteria, with no binding observed for Gram-negative bacterial species [23, 24]. Furthermore, vancomycin-based optical tracers have been shown to bind to clinical vancomycin-resistant *vanA*- or *vanB*-positive *Enterococcus faecium* isolates, indicating that vancomycin resistance does not necessarily preclude tracer accumulation [21, 23].

Future research should focus on gaining a better understanding of the dynamic interactions between vancomycin-based tracers and bacteria in the infectious niche. Additionally, it will be important to optimise the tracer kinetics to enhance tracer accumulation at sites of infection and, if possible, to improve the bacterial detection with vancomycin-based PET tracers by the implementation of radionuclides with a longer half-life than that of ^18^F. By building on the promising results in the applied murine K-wire infection model, we believe that further development of vancomycin-based tracers towards clinical translation holds great promise for bacteria-targeted infection imaging, using both FLI and PET.

## Conclusion

In this study, vancomycin-based fluorescence imaging and PET imaging were explored in a murine model of orthopaedic trauma implant infection, providing the first proof-of-principle application of these tracers in in vivo orthopaedic biofilm infection imaging. Fluorescence imaging with vancomycin-800CW reliably distinguished *S. aureus* infection from *E. coli* infection and sterile conditions. Vancomycin-based PET tracers demonstrated higher uptake in infected tissue than in controls, but these tracers did not distinguish between the *S. aureus* and *E. coli* infections. We hypothesize that the latter could be related to a combination of relatively low bacterial loads and slow tracer clearance, or to non-specific (blood-pool) accumulation of the tracer due to capillary leakage and vasodilation associated with an inflammatory response. Altogether, our findings support further investigation into the application potential of vancomycin-based tracers for non-invasive imaging of prosthetic joint- and fracture-related infections.

## Supplementary Information

Below is the link to the electronic supplementary material.

Supplementary Material 1

Supplementary Material 2

## Data Availability

All data are provided with the manuscript.
