# Supplementary material for: In vivo bacteria-targeted imaging with vancomycin-based positron emission tomography and optical tracers in an orthopaedic trauma implant infection model
Source: Eur J Nucl Med Mol Imaging. 2026 Jun 19;53(10):6098–110. doi: 10.1007/s00259-026-07997-x (PMC13421359; doi:10.1007/s00259-026-07997-x)
Supplement: Supplementary file 2 — Supplementary Material 2 [file 259_2026_7997_MOESM2_ESM.pdf]

Supplemental Materials

In Vivo Bacteria-Targeted Imaging with Vancomycin-Based Positron Emission Tomography and Optical Tracers in an Orthopaedic Trauma Implant Infection Model

G.B. Spoelstra[1,*], L.M. Braams[2,*], F.F.A. IJpma[3], S. Piersma[2], C.S. Braams[2], N.M. Bernthal[4], K.P. Francis[4,], A.W.J.M. Glaudemans[1], M. van Oosten[2], B.L. Feringa[5], W. Szymanski[6,7], P.H. Elsinga[1,†] and J.M. van Dijl[2,†,#]

[1] University of Groningen, University Medical Center Groningen, Department of Nuclear Medicine and Molecular Imaging, Hanzeplein 1, 9713GZ Groningen, The Netherlands;

[2] University of Groningen, University Medical Center Groningen, Department of Medical Microbiology and Infection Prevention, Hanzeplein 1, 9713GZ Groningen, The Netherlands;

[3] University of Groningen, University Medical Center Groningen, Department of Trauma Surgery, Hanzeplein 1, 9713GZ Groningen, The Netherlands;

[4] University of California, Department of Orthopaedic Surgery, Los Angeles, CA 90095, USA;

[5] Stratingh Institute for Chemistry, University of Groningen, Nijenborgh 4, 9747AG Groningen

[6] University of Groningen, University Medical Center Groningen, Department of Radiology, Hanzeplein 1, 9713GZ Groningen, The Netherlands;

[7] University of Groningen, Groningen Research Institute of Pharmacy, Department of Medicinal Chemistry, Photopharmacology and Imaging, Antonius Deusinglaan 1, 9713AV Groningen, The Netherlands

[*,†] equal contributions

# Corresponding author: Prof. dr. Jan Maarten van Dijl, ORCID ID 0000-0002-5688-8438; Hanzeplein 1, 9700RB Groningen, The Netherlands; email: j.m.van.dijl01@umcg.nl

Table of Content

- **Figure S1**: Chemical structures of vancomycin-IRDye800CW, [^{18}F]VE1-PQ-vancomycin and [^{18}F]BODIPY-FL-vancomycin
- **Figure S2:** ROI placement methodology for PET/CT image analysis
- **Figure S3**: Colony-forming units (CFUs) in bacterial inoculums and harvested tissues post-termination
- **Figure S4**: Scanning electron microscopy of biofilm formation on K-wire implants
- **Figure S5**: Optimisation of post-injection intervals for vancomycin-based imaging tracers
- **Figure S6**: Biodistribution of vancomycin-800CW
- **Figure S7**: Two-dimensional representation of the three-dimensional visualisation of PET/CT imaging with [^{18}F]BODIPY-FL-vancomycin
- **Figure S8**: Biodistribution of [^{18}F]BODIPY-FL-vancomycin, [^{18}F]VE1-PQ-vancomycin and [^{18}F]FDG
- **Table S1**: Biodistribution of [^{18}F]BODIPY-FL-vancomycin, [^{18}F]VE1-PQ-vancomycin and [^{18}F]FDG
- **Table S2:** Tracer Molecules/CFU
- **Movie S1**: Three-dimensional visualisation of PET/CT imaging with [^{18}F]BODIPY-FL-vancomycin

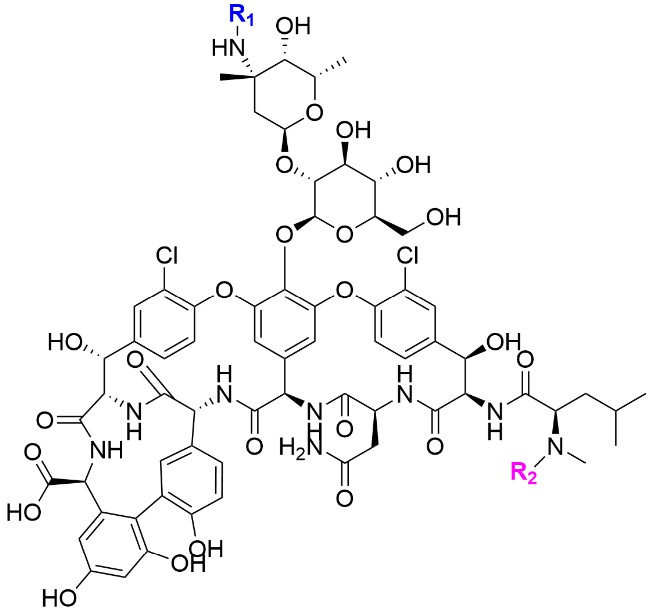

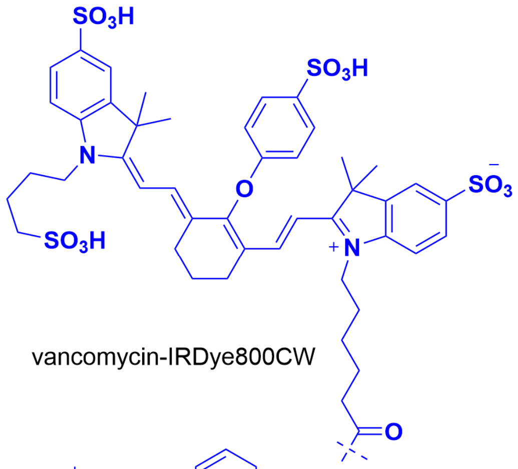

vancomycin-IRDye800CW

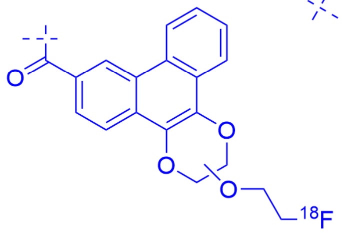

[^{18}F]VE1-PQ-vancomycin

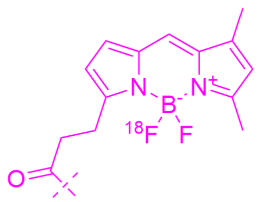

[^{18}F]BODIPY-FL-vancomycin

Figure S1: Chemical structures of vancomycin-IRDye800CW, [^{18}F]VE1-PQ-vancomycin and [^{18}F]BODIPY-FL-vancomycin. IRDye800CW and [^{18}F]VE1-PQ are conjugated to the primary amine (R_1) of vancomycin, whilst [^{18}F]BODIPY-FL is conjugated to the secondary amine (R_2) of vancomycin.

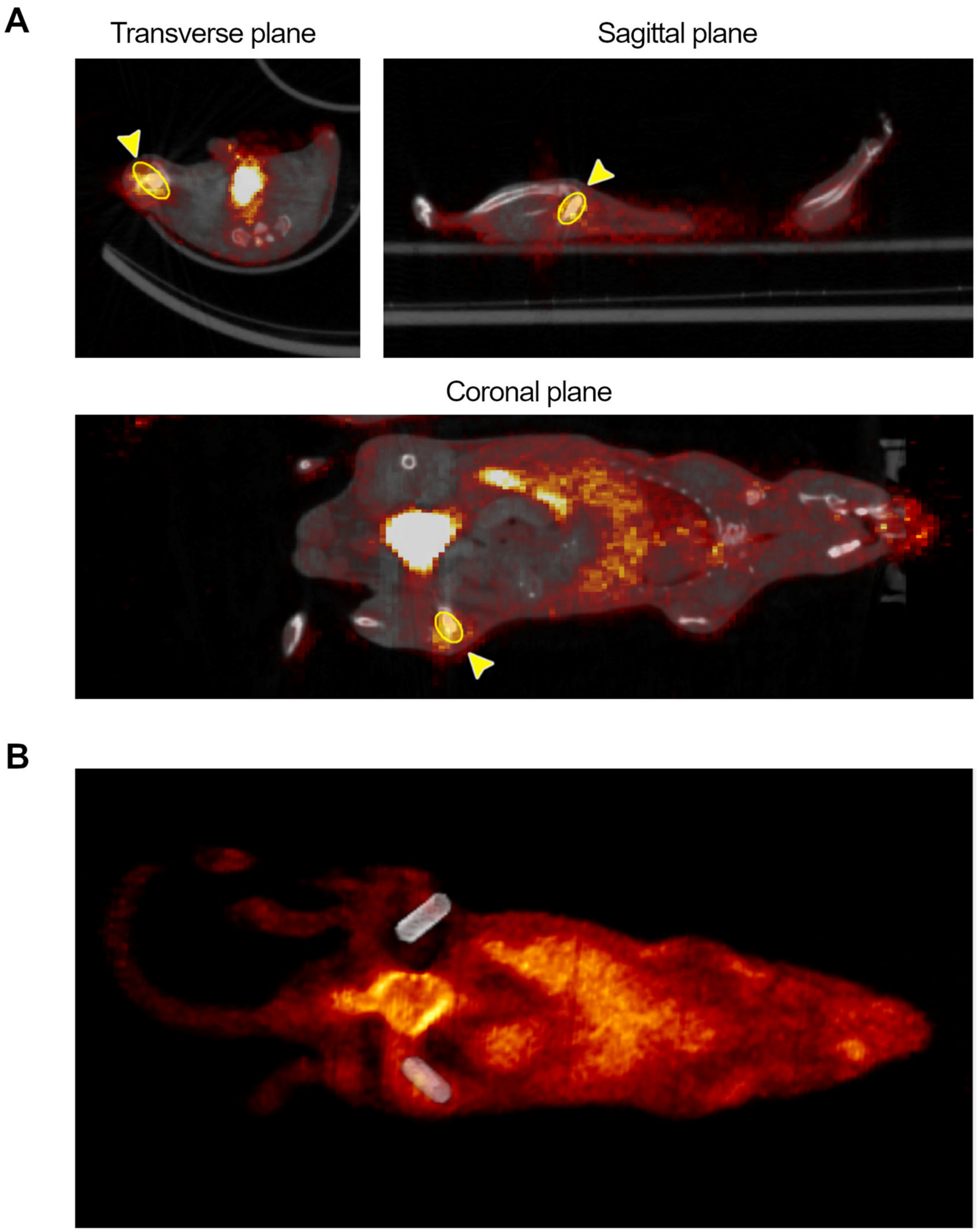

Figure S2: ROI placement methodology for PET/CT image analysis, performed in AMIDE (v1.0.6). **(A)** Representative transverse, coronal, and sagittal views of a PET/CT scan from a single *S. aureus*-infected animal imaged with [^{18}F]BODIPY-FL-vancomycin. A cylindrical ROI (⌀ 3 mm, length 10 mm) was placed over the K-wire location in the left femur, referencing the CT scan for anatomical guidance. Yellow arrow heads mark the positions of the ROIs. The differing outline shapes across views reflect the three-dimensional cylindrical geometry. **(B)** Three-dimensional PET rendering of the same animal, showing the two ROIs that were drawn per animal: one ROI covering the K-wire-implanted femur and one ROI covering the contralateral control femur without an implanted K-wire (light grey cylinders).

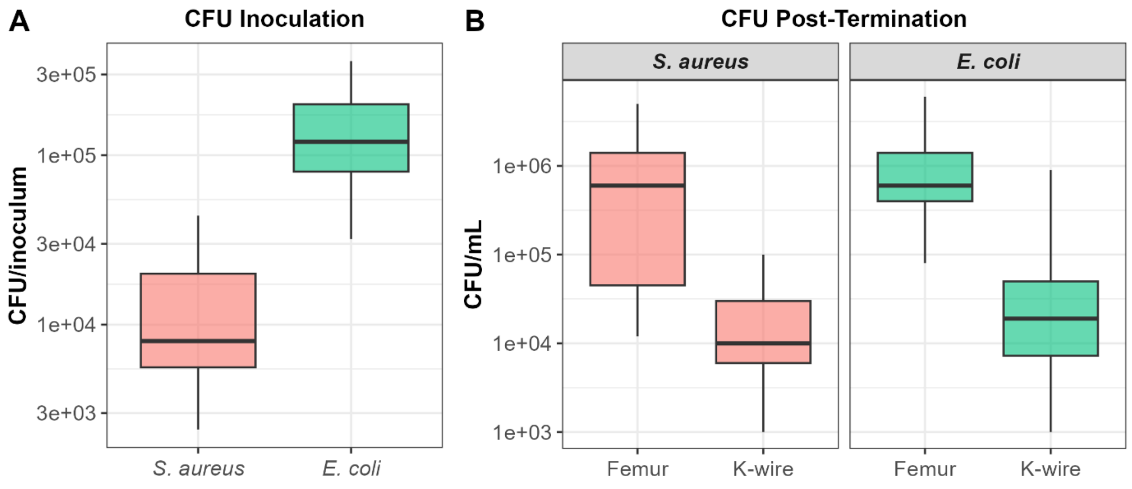

Figure S3: Colony-forming units (CFUs) in bacterial inoculums and harvested tissues post-termination. Data is represented as the median with interquartile range (IQR). **(A)** CFUs at the time of inoculation. From the bacterial inoculum, a dilution series was prepared for CFU determination. Bacterial loads were set to approximate 10^4 CFUs per inoculum (in 2.5 µL) for *S. aureus*, and 10^5 CFU per inoculum (in 2.5 µL) for *E. coli*. The y-axis is logarithmically scaled. **(B)** CFU counts post-termination. Femur, tibia and K-wire were collected from the infected leg after termination of the animals. Subsequently, the femur and tibia were homogenised using a tissue homogeniser, and the cellular debris was pelleted by centrifugation. The K-wire was placed in 500 µL MHB + 0.3% Tween solution and vortexed for 5 min to disrupt the biofilm from the surface. From the tissue homogenate and K-wire supernatant, CFUs were determined and expressed as CFU/mL tissue. Of note, the tibia was typically culture negative.

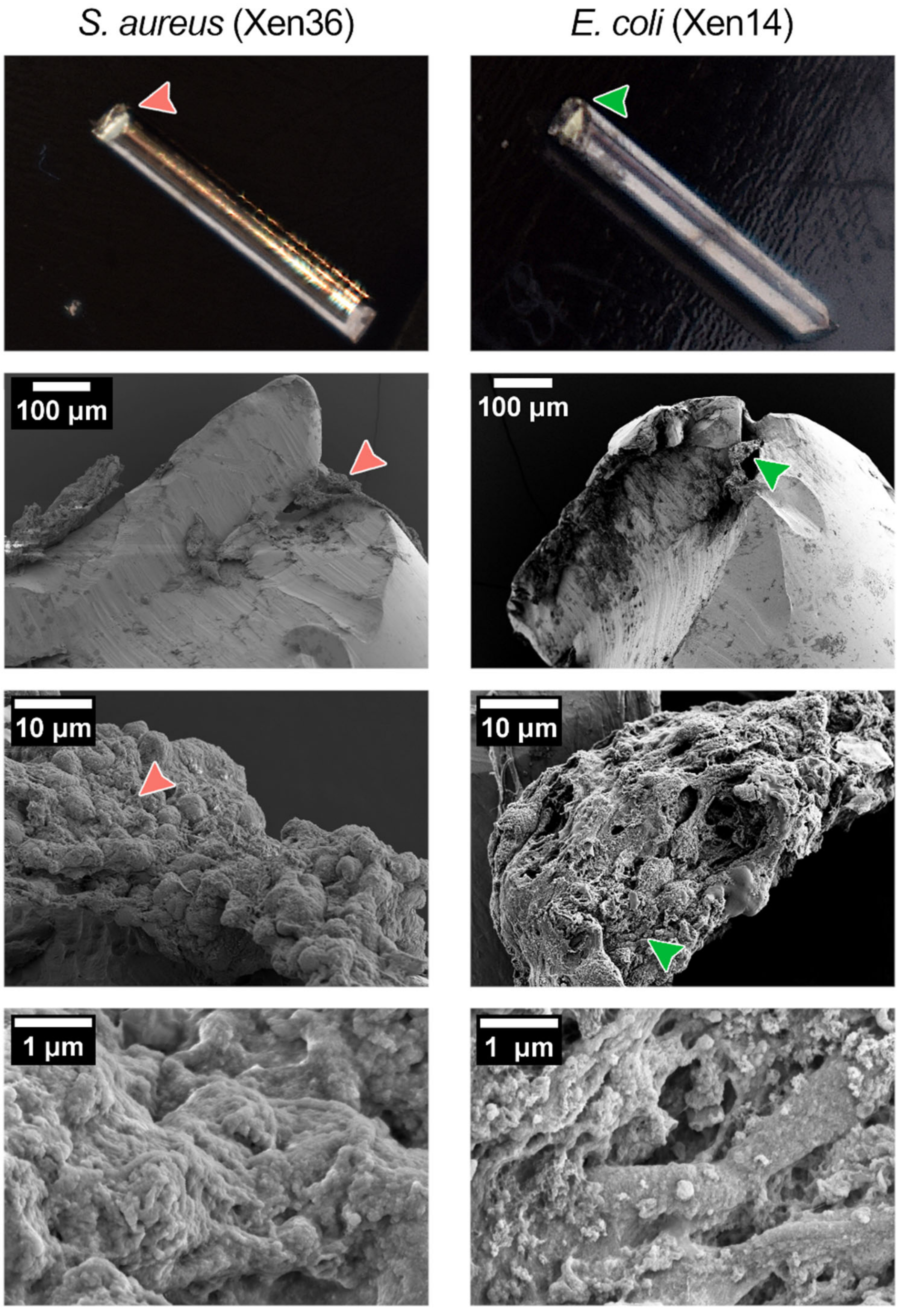

Figure S4: Scanning electron microscopy of biofilm formation on K-wire implants. Representative white light and scanning electron microscopy (SEM) images of the intra-articular portion of the K-wire implant. The upper row presents white light images (upper two panels), followed by subsequent SEM images at low (scale bars: 100 µm) and high magnifications (scale bars: 10 µm and 1 µm). Red arrows (*S. aureus* biofilm) and green arrows (*E. coli* biofilm) mark the areas where magnified views were captured. Images are shown for K-wire implants from one mouse per investigated group (n = 2), with consistent findings. Bacteria are embedded in a distinctive polymeric matrix, characteristic of bacterial biofilms.

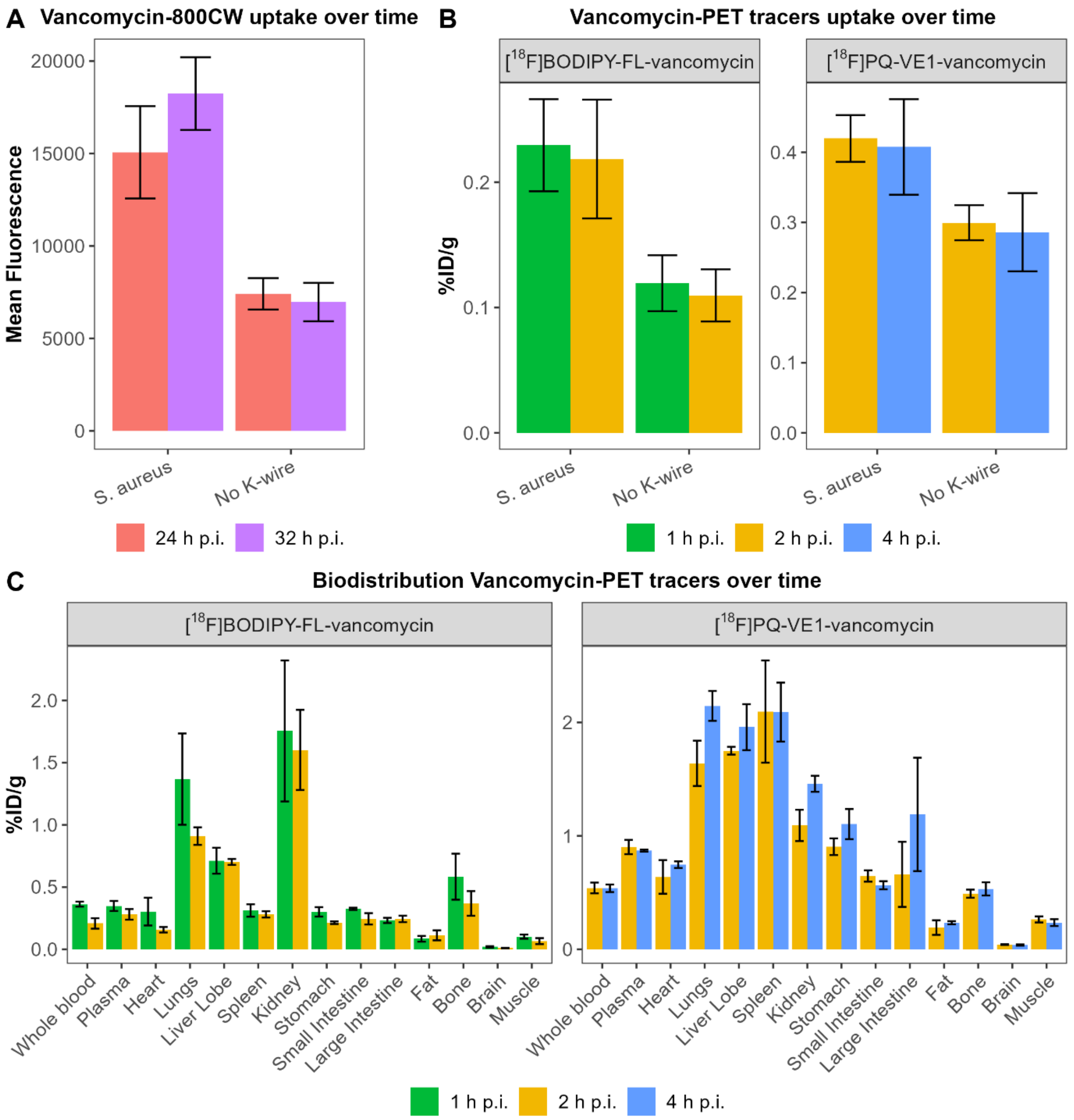

Figure S5: Optimisation of post-injection (p.i.) intervals for vancomycin-based imaging tracers. Pilot studies using an *S. aureus* K-wire infection model (n = 6 per tracer) evaluated two p.i. intervals per tracer (n = 3 each) to determine optimal imaging timing. **(A)** For vancomycin-800CW, previously imaged at 24 h p.i. (17), comparison with 32 h p.i. (red vs. purple) showed higher uptake at the infection site at 32 h, which was therefore selected for subsequent experiments. **(B)** [^{18}F]BODIPY-FL-vancomycin and [^{18}F]VE1-PQ-vancomycin, both previously imaged at 1 h p.i. (22), were tested at longer intervals because of their distinct pharmacokinetics. Due to the faster renal clearance, the [^{18}F]BODIPY-FL-vancomycin tracer was assessed at 1 h (green) and 2 h p.i. (yellow), while the [^{18}F]VE1-PQ-vancomycin tracer was tested at 2 h (yellow) and 4 h p.i. (blue). **(C)** Uptake at the infection site and biodistribution data revealed minimal benefit from delayed imaging at these intervals. As a result, 1 h p.i. was chosen for imaging with [^{18}F]BODIPY-FL-vancomycin and 2 h p.i. for imaging with [^{18}F]VE1-PQ-vancomycin in subsequent studies. Data are shown as mean ± standard deviation (SD).

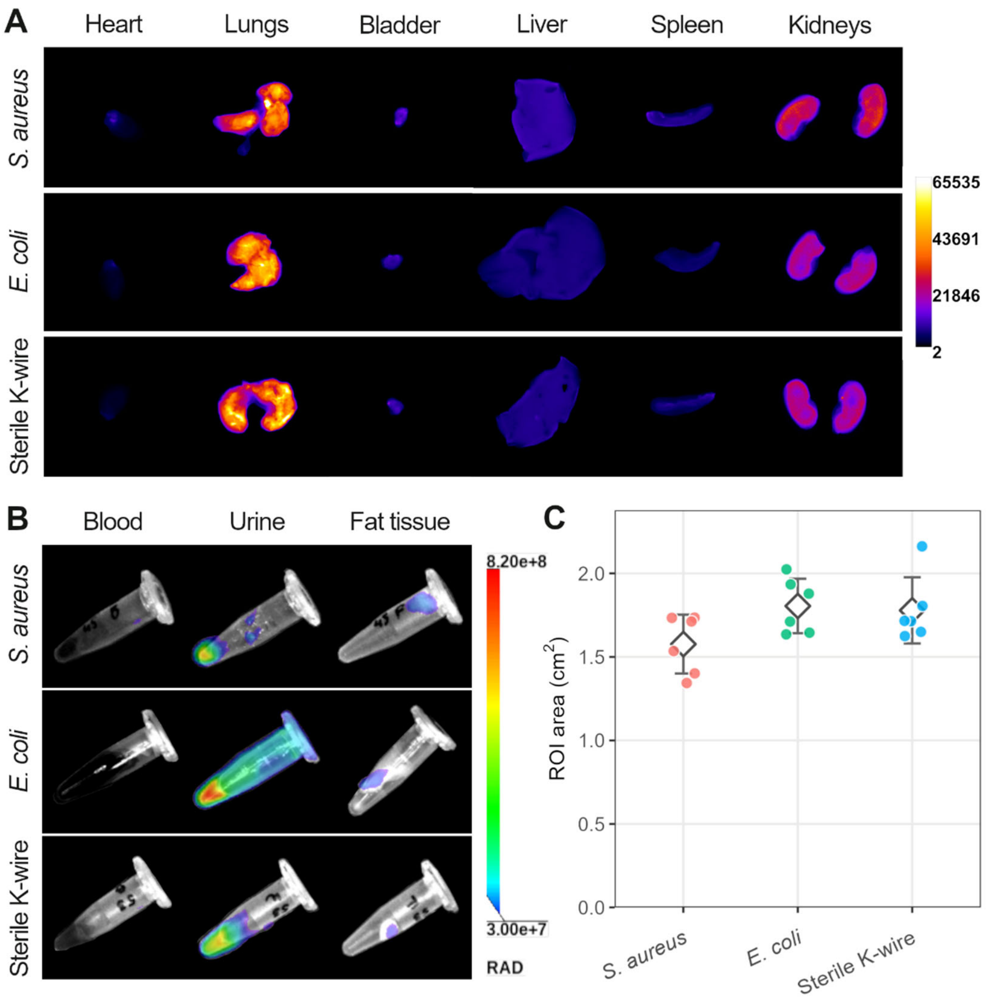

Figure S6: Biodistribution of vancomycin-800CW. (**A**) Representative fluorescence images of relevant organs and tissues acquired using a Typhoon Biomolecular Imaging System at 32 h post-injection (n = 1 of 6 per group; comparable results across animals). High signal accumulation is observed in the lungs and kidneys in all groups. The scale indicates the fluorescence intensity. (**B**) Corresponding tissue fluorescence images captured with the LAGO imaging system. Fluorescence signal is consistently detected in urine across groups. The scale indicates radiance. **(C)** Average ROI areas drawn over the hind legs of mice with an implanted K-wire across experimental conditions. No significant differences in ROI sizes were observed between groups (Welch's ANOVA: $F(2, 9.94) = 2.87$, $p = 0.104$).

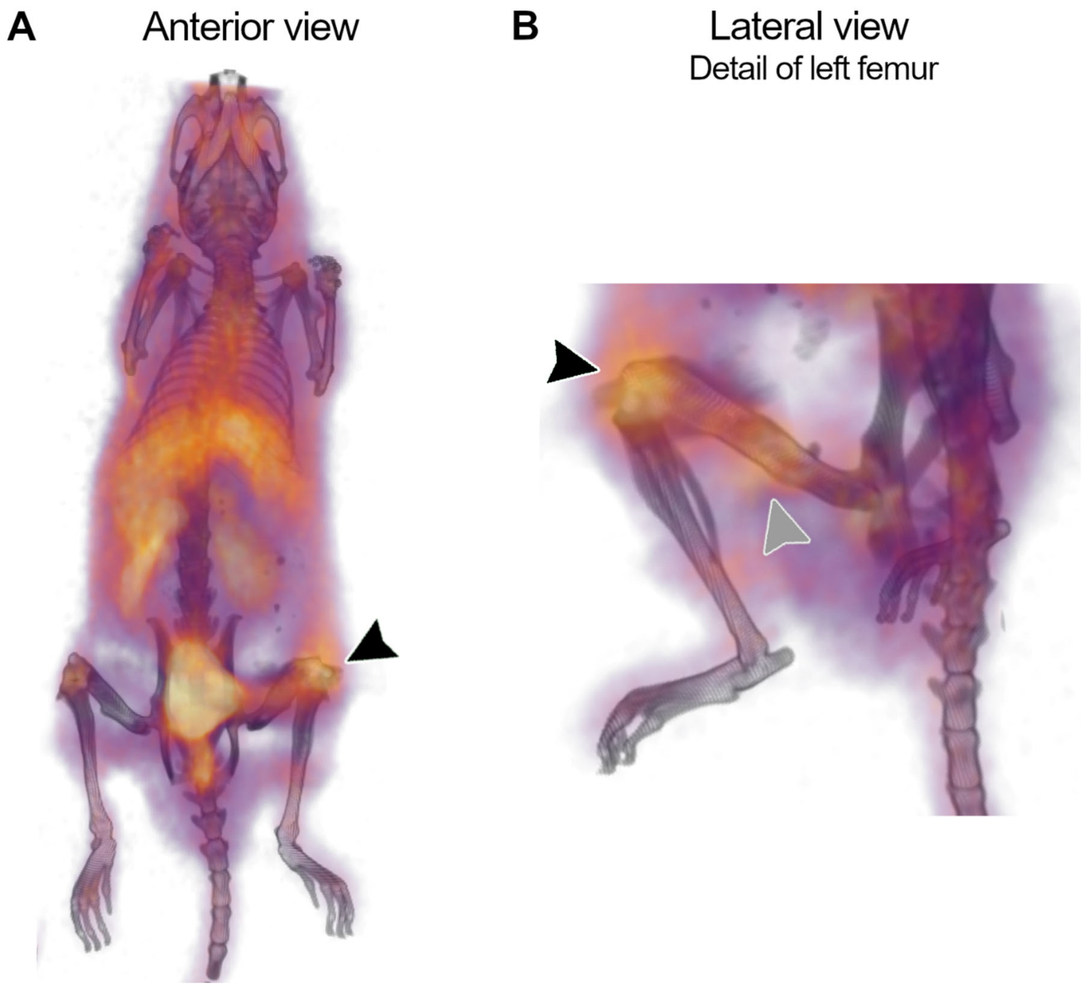

Figure S7: Two-dimensional representation of the three-dimensional visualisation of PET/CT imaging with [^{18}F]BODIPY-FL-vancomycin. **(A)** Anterior view of [^{18}F]BODIPY-FL-vancomycin-based imaging of a K-wire infection by *S. aureus* (black arrow head, left knee-joint space). The animal is in supine position. The image was generated using the open-source Python tool PyVIVIPET, data is normalized. **(B)** Close-up lateral/dorsal view of the infected K-wire. Signal accumulation is visible at the distal end of the K-wire within the knee-joint space (black arrow head) and around the proximal end in the femoral shaft (grey arrow head). Bladder signal was masked in panel (B) to provide a clearer view of tracer uptake in the leg. Supplemental Movie S1 visualizes the present PET/CT with [^{18}F]BODIPY-FL-vancomycin in three dimensions.

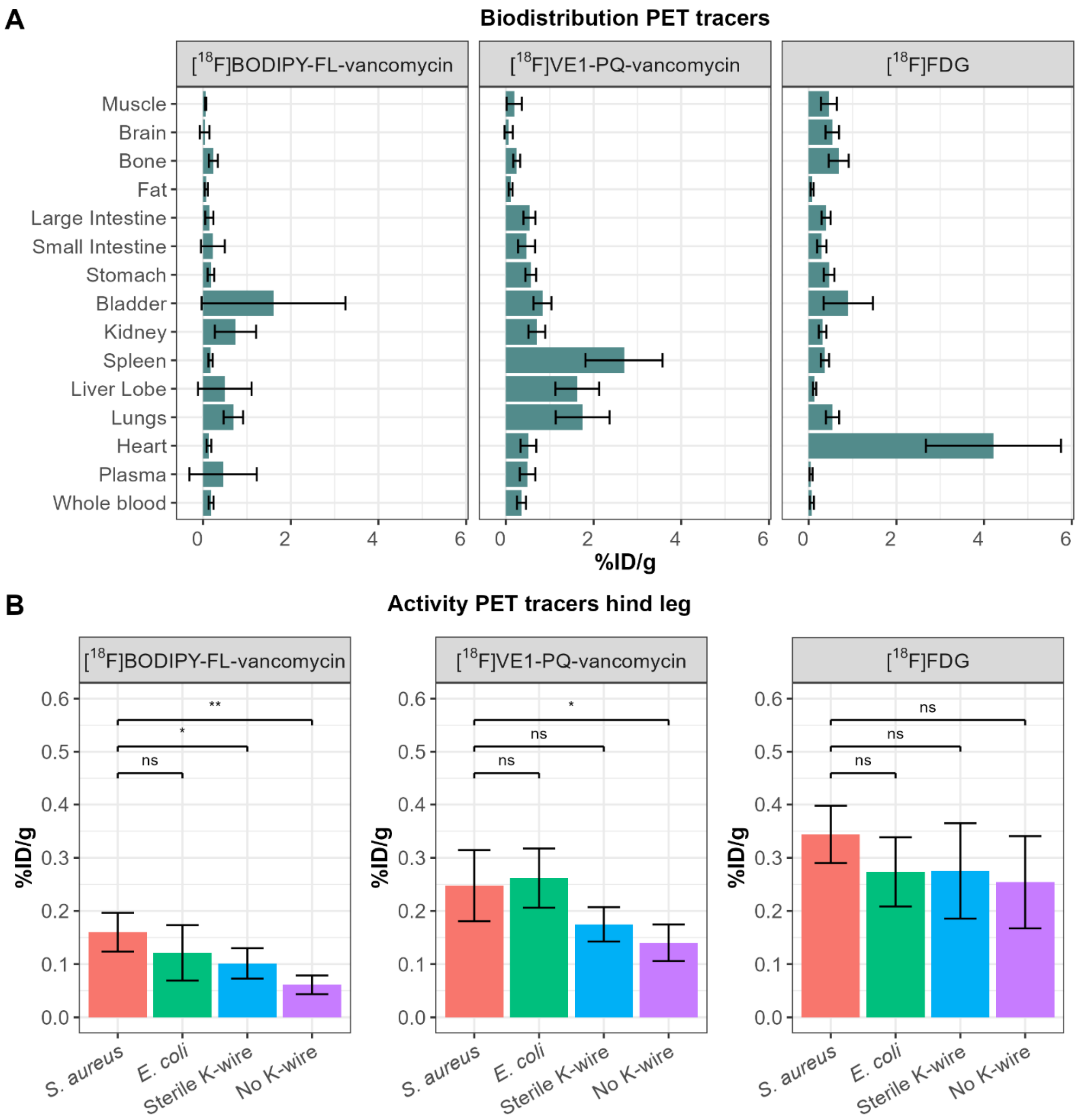

Figure S8: Biodistribution of [^{18}F]BODIPY-FL-vancomycin, [^{18}F]VE1-PQ-vancomycin and [^{18}F]FDG. **(A)** Biodistribution analysis of the PET tracers (n = 18 per tracer). The radioactivity was measured as the percentage of the injected dose per gram (%ID/g) of tissue, expressed as mean ± SD. **(B)** Uptake of both ^{18}F-vancomycin-based tracers and [^{18}F]FDG in the hind legs (n = 6 per condition per tracer). The x-axis represents the infection status of the legs with *S. aureus*- or *E. coli*-induced K-wire infection, PBS-treated sterile K-wires, or healthy control tissues (no K-wire) from the contralateral legs. Data is represented as % ID/g, expressed as the mean ± SD. Welch's ANOVA with planned Welch's t-tests versus *S. aureus* (Holm-adjusted): *, $p < 0.05$; **, $p < 0.01$; ns, not significant.

Table S1: Biodistribution of [^{18}F]BODIPY-FL-vancomycin, [^{18}F]VE1-PQ-vancomycin and [^{18}F]FDG

	[^{18}F]BODIPY-FL-vancomycin		[^{18}F]VE1-PQ-vancomycin		[^{18}F]FDG	
**Sample**	**% ID/g**	**SD**	**% ID/g**	**SD**	**% ID/g**	**SD**
Whole blood	0.18	0.05	0.36	0.10	0.07	0.05
Plasma	0.46	0.77	0.49	0.18	0.05	0.03
Heart	0.13	0.05	0.51	0.18	4.21	1.54
Lungs	0.69	0.22	1.75	0.61	0.54	0.15
Liver Lobe	0.49	0.61	1.63	0.50	0.14	0.04
Spleen	0.17	0.05	2.69	0.88	0.37	0.09
Kidney	0.74	0.47	0.71	0.19	0.32	0.09
Bladder	1.61	1.64	0.83	0.21	0.90	0.56
Stomach	0.18	0.07	0.57	0.12	0.46	0.12
Small Intestine	0.22	0.27	0.47	0.19	0.30	0.11
Large Intestine	0.14	0.09	0.54	0.14	0.40	0.10
Fat	0.07	0.04	0.11	0.04	0.08	0.03
Bone	0.23	0.10	0.25	0.08	0.68	0.23
Brain	0.03	0.11	0.06	0.09	0.54	0.15
Muscle	0.06	0.01	0.19	0.17	0.46	0.18

ID, injected dose; SD, standard deviation.

Table S2. Tracer Molecules/CFU. Estimated number of tracer molecules per colony-forming unit (CFU) in the femur of mice with implant-associated infection, calculated from gamma counter-derived %ID/g, net injected dose, molar activity, and post-termination femur homogenate CFU counts.

Tracer	Condition	$\log_{10}$(Molecules/CFU) Mean ± SD
[^{18}F]BODIPY-FL-vancomycin	*S. aureus*	5.10 ± 0.31
	E. coli	5.35 ± 0.20
[^{18}F]VE1-PQ-vancomycin	*S. aureus*	3.96 ± 0.66
	E. coli	3.55 ± 0.33

CFU, colony forming units; SD, standard deviation.
